# Exercise-induced atrioventricular block in a patient with a pacemaker: a functional consequence of AV search algorithms—a case report

**DOI:** 10.1093/ehjcr/ytag244

**Published:** 2026-03-24

**Authors:** Clara Nuevo-Gallardo, José Miguel Rojo-Pérez, Luis Javier Doncel-Vecino, Lorenzo Muñoz-Santos, Antonio Chacón-Piñero

**Affiliations:** Cardiology Department, University Hospital of Badajoz, Avenida de Elvas s/n, Badajoz 06006, Spain; Cardiology Department, University Hospital of Badajoz, Avenida de Elvas s/n, Badajoz 06006, Spain; Cardiology Department, University Hospital of Badajoz, Avenida de Elvas s/n, Badajoz 06006, Spain; Arrythmia Unit, Cardiology Department, University Hospital of Badajoz, Avenida de Elvas s/n, Badajoz 06006, Spain; Arrythmia Unit, Cardiology Department, University Hospital of Badajoz, Avenida de Elvas s/n, Badajoz 06006, Spain

**Keywords:** Atrioventricular block, DDD pacemaker, Atrioventricular search algorithm, Post-ventricular atrial refractory period, Exercise testing, Case report

## Abstract

**Background:**

Algorithms designed to promote intrinsic atrioventricular (AV) conduction in dual-chamber pacemakers aim to reduce unnecessary right ventricular pacing and its long-term adverse effects. The AV Search+ algorithm periodically prolongs the AV delay to facilitate native conduction and is particularly useful in non–pacemaker-dependent patients. However, under certain conditions, such algorithms may lead to unexpected disturbances in AV synchrony, especially during exercise.

**Case summary:**

A 65-year-old woman with symptomatic second-degree AV block (Mobitz II) underwent dual-chamber pacemaker implantation. During follow-up, she developed progressive exertional dyspnoea. Exercise stress testing revealed a paradoxical decrease in heart rate during increasing workload with reproduction of symptoms. Programmer-monitored exercise testing demonstrated a functional 2:1 AV block caused by atrial undersensing, as atrial depolarizations occurred within the post-ventricular atrial refractory period during AV Search+–mediated AV delay prolongation. Deactivation of the AV Search+ algorithm restored stable 1:1 AV conduction during exercise and resulted in complete symptom resolution.

**Conclusion:**

This case highlights a rare but clinically relevant complication of AV conduction search algorithms in dual-chamber pacemakers. Although these algorithms effectively reduce ventricular pacing, inappropriate interactions between prolonged AV delays and atrial refractory periods may result in symptomatic functional AV block, particularly during exercise and in patients with advanced AV conduction disease. Exercise testing with device interrogation is a key diagnostic tool in pacemaker patients with exertion-related symptoms, and individualized device programming is essential to ensure optimal clinical outcomes.

Learning points

- AV conduction search algorithms can paradoxically induce symptomatic 2:1 functional atrioventricular block during exercise in pacemaker patients.
- Exercise-induced symptoms in paced patients should prompt evaluation for device–algorithm interactions using exercise testing with device interrogation.
- High ventricular pacing burden limits the benefit of AV search algorithms.

## Introduction

Atrioventricular block (AVB) and sinus node dysfunction are currently the most common indications for permanent pacemaker implantation, particularly in elderly patients. Despite the undisputed clinical benefits of permanent pacing, the physiological consequences of chronic right ventricular (RV) pacing have been increasingly recognized over recent decades.^1,2^ Pacing-induced cardiomyopathy is a well-established complication of chronic RV pacing and is characterized by progressive left ventricular systolic dysfunction. Its reported prevalence ranges from 6% to 25%, depending on patient characteristics and the percentage of ventricular pacing, and it is associated with increased heart failure hospitalizations and cardiovascular mortality.^2,3^ Consequently, a major goal of modern device programming is to minimize unnecessary ventricular pacing while preserving adequate atrioventricular (AV) synchrony.^3,4^

To address this issue, device manufacturers have developed algorithms aimed at promoting intrinsic AV conduction.^5–9^ In dual-chamber pacemakers, the AV Search+ algorithm (Boston Scientific) periodically prolongs the AV interval to detect native conduction and reduce ventricular pacing. This strategy is particularly beneficial in non-pacemaker-dependent patients with intermittent AV conduction and an expected low percentage of ventricular pacing.^5^ However, AV conduction search algorithms are not free from adverse effects. Data regarding their haemodynamic and arrhythmic consequences during exercise are limited, and under certain conditions, particularly at high sinus rates, the interaction between prolonged AV delays and atrial refractory periods may result in unexpected disturbances of AV synchrony.^4,10^ These phenomena may be absent at rest and therefore underrecognized during routine follow-up.

We report a case of symptomatic functional 2:1 AV block during exercise caused by a frequency-dependent interaction between the AV Search+ algorithm and atrial refractory periods in a dual-chamber pacemaker. The novelty of this case lies in the clear reproduction of symptoms during exercise testing, their direct correlation with intracardiac electrograms, and the demonstration that device programming, rather than progression of conduction disease, was the underlying mechanism. This case highlights the importance of appropriate patient selection for AV conduction search algorithms and underscores the diagnostic value of exercise testing in pacemaker patients with exertion-related symptoms.

## Summary figure

**Table:** 

2017	Implantation of a dual-chamber DDD permanent pacemaker for symptomatic second-degree Mobitz II atrioventricular block.
2017–2022	Dyspnoea on moderate exertion. Exercise test with an accelerated chronotropic curve, adjusting pacemaker parameters and starting rate-controlling medication. Stress echocardiogram negative for ischaemia.
April 2025	Increased exertional dyspnoea reported during follow-up visits, with a new exercise test requested.
May 2025	A stress test monitored with the pacemaker programmer demonstrated an effective 2:1 AV block, caused by P-wave sensing within the post-ventricular atrial refractory period (PVARP) during intrinsic AV conduction search. The algorithm was deactivated, leading to symptomatic improvement.
June 2025	Asymptomatic from a cardiovascular standpoint.

## Case presentation

A 65-year-old woman with no known cardiovascular risk factors presented to the emergency department with a two-week history of exertional dyspnoea. She had no prior history of ischaemic heart disease; her only relevant medical condition was gastro-oesophageal reflux disease.

On admission, a 12-lead electrocardiogram revealed second-degree AVB type Mobitz II. She was admitted to the cardiology department, and permanent pacemaker implantation was indicated. A dual-chamber pacemaker was implanted without complications. Chest radiography confirmed correct lead positioning, and the patient was discharged 24 h after the procedure.

Initial follow-up visits showed appropriate device function and satisfactory clinical evolution. However, the patient later reported dyspnoea during moderate exertion, prompting further evaluation. An exercise stress test demonstrated an accelerated chronotropic response with abrupt termination of heart rate increase at the programmed maximum tracking rate. Device reprogramming was performed, and rate-limiting pharmacological therapy was initiated. A subsequent stress echocardiogram was negative for inducible myocardial ischaemia. The patient remained clinically stable for a period thereafter.

**Figure 1:**
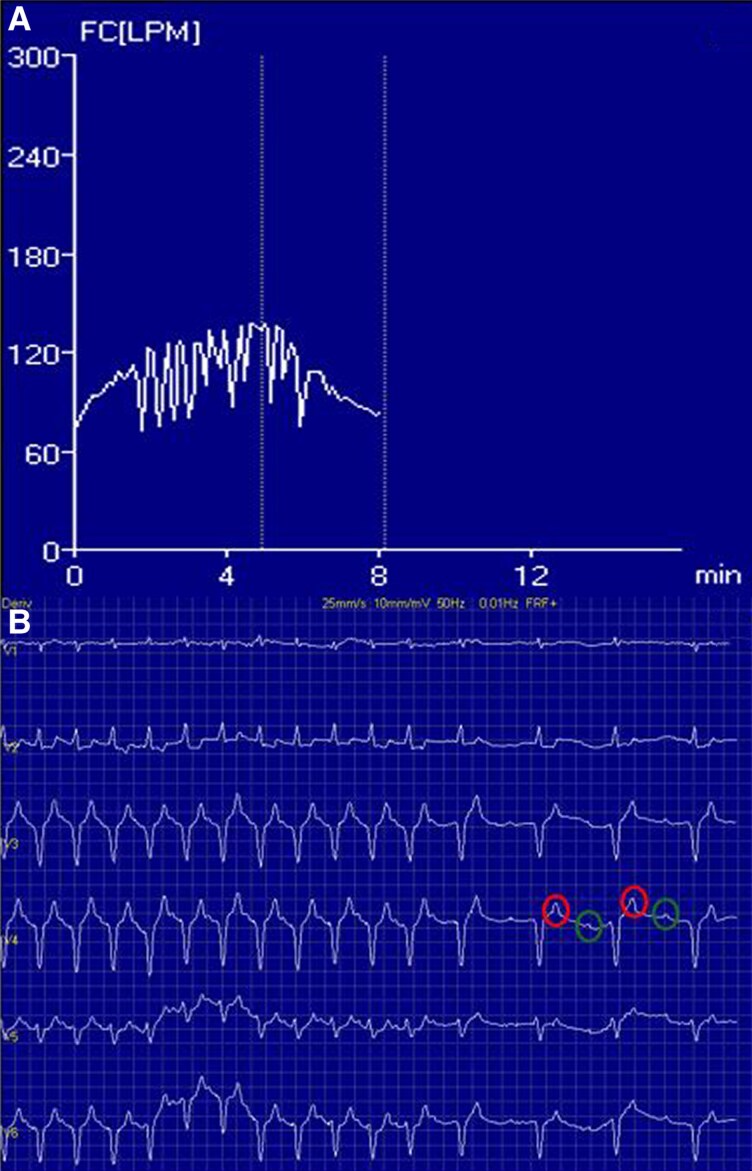
Paroxysmal atrioventricular block during exercise testing. *A*) Heart rate curve showing a paradoxical decrease as exercise progresses. *B*) Surface electrocardiogram demonstrating paroxysmal atrioventricular block. Initially, sinus tachycardia at 130 bpm is observed. Following activation of the AV Search+ algorithm, AV interval prolongation is observed, with atrial depolarizations followed by paced QRS complexes (green circles) alternating with non-tracked P waves which fall within the PVARP (red circles).

At a later outpatient visit, she reported progressive worsening of exertional dyspnoea. Repeat exercise testing showed a paradoxical drop in heart rate with increasing workload, accompanied by episodes of bradycardia consistent with 2:1 AV block and reproduction of symptoms (*Figure 1*). The test was interrupted and repeated under direct monitoring with the pacemaker programmer.

**Figure 2:**
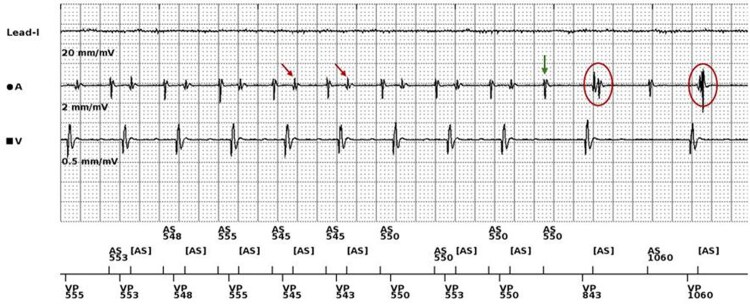
Atrial and ventricular electrogram recordings during exercise testing. Initial atrial sensing (AS) with ventricular pacing (VP) at approximately 100 bpm is observed. An additional atrial sensing signal immediately after ventricular pacing corresponds to ventricular far-field sensing (red arrows). Activation of the AV Search+ algorithm results in AV interval prolongation (green arrow), followed by a paced ventricular event. Finally, it is seen how ventricular far-field sensing overlaps with the intrinsic P wave, which falls within the post-ventricular atrial refractory period (PVARP) and is therefore not tracked by the device (red circles).

During programmer-monitored exercise testing, ventricular far-field sensing was observed in the atrial channel, overlapping with atrial depolarization. Prolongation of the AV interval induced by the AV Search+ algorithm caused subsequent *P* waves to fall within the post-ventricular atrial refractory period (PVARP), resulting in atrial undersensing and abrupt exercise-related bradycardia due to functional 2:1 AV block (*Figure 2*). The AV Search+ algorithm was disabled, restoring stable 1:1 AV conduction during exercise and leading to marked symptomatic improvement.

At follow-up, the patient remained asymptomatic, maintaining an active lifestyle and experienced no further device-related complications.

## Discussion

Chronic RV pacing may adversely affect left ventricular systolic function by inducing electrical and mechanical dyssynchrony, ultimately leading to pacing-induced cardiomyopathy. Although definitions vary across studies, a commonly accepted definition includes a left ventricular ejection fraction ≤50% with a reduction greater than 10% from baseline in the presence of a ventricular pacing burden exceeding 20%.^1–3^

To reduce unnecessary ventricular pacing, modern devices incorporate algorithms that promote intrinsic AV conduction whenever possible.^4–9^ Among these, the AV Search+ algorithm periodically prolongs the AV delay in dual-chamber pacemakers implanted in non-pacemaker-dependent patients, aiming to facilitate intrinsic AV conduction and reduce ventricular pacing burden.^4,5^

When activated, AV Search+ prolongs the AV delay for up to eight consecutive cardiac cycles and maintains this extended delay as long as intrinsic PR intervals remain shorter than the programmed search AV interval. The baseline AV delay is restored once the search period ends without intrinsic ventricular sensing or when two paced ventricular events occur within a rolling 10-cycle window.^4,10^

Despite its benefits, AV Search+ may be associated with adverse effects. Pacemaker-mediated tachycardia and functional AV block have been described, although reports are limited.^4^ Functional 2:1 AV block during exercise may occur due to atrial undersensing when prolonged AV delays interact with atrial refractory periods during sinus tachycardia, causing *P* waves to fall within the PVARP and resulting in an abrupt reduction in ventricular rate.^4,9,10^

The PVARP is the interval following a ventricular sensed or paced event during which atrial signals are ignored, protecting against inappropriate tracking of retrograde atrial activity, and the total atrial refractory period consists of the AV interval plus the PVARP. The heart rate at which functional 2:1 AVB develops depends on the duration of this total refractory period, which may be substantially prolonged during intrinsic conduction search.^4,10^

Adjustment of atrial refractory parameters such as PVARP or the post-ventricular atrial blanking period (PVAB) may represent a reasonable alternative in selected patients with preserved intrinsic AV conduction. However, AV conduction search algorithms are not appropriate in patients with advanced or permanent high-grade AV block, such as Mobitz type II, particularly when ventricular pacing dependency is present.^4,5^ In our patient, symptom recurrence was associated with a high ventricular pacing burden, indicating progression of conduction disease and loss of the intended benefit of AV Search+. In this setting, prolonged AV delays directly contributed to functional AV block during exercise, and isolated adjustment of atrial refractory parameters would not have reliably prevented recurrence while the algorithm remained active.

Compared with previously reported cases, this report is unique in that symptoms were clearly reproduced during exercise testing and directly correlated with intracardiac electrograms and real-time device behaviour, allowing definitive identification of a programming-related functional AV block rather than intrinsic conduction deterioration.

From a practical standpoint, this case emphasizes the importance of considering device-related mechanisms in pacemaker patients with exertion-related symptoms. Exercise testing with device interrogation should be strongly considered in this setting, as it may reveal frequency-dependent AVB related to programming algorithms and guide appropriate device optimization.^11,12^

Moreover, with the increasing adoption of physiological pacing strategies such as His bundle pacing or left bundle branch area pacing, which minimize ventricular dyssynchrony, the indication for AV conduction search algorithms should be reconsidered on an individual basis, balancing their theoretical benefits against potential adverse effects, particularly in patients with established ventricular pacing dependence.

## Conclusions

This case illustrates an uncommon but clinically relevant complication of the AV Search+ algorithm, resulting in symptomatic functional AV block during exercise due to frequency-dependent atrial undersensing.

Although atrial refractory periods occur after both sensed and paced ventricular events, functional AV block is more likely during ventricular pacing with prolonged AV delays, particularly in patients with advanced AV conduction disease.

Careful follow-up and individualized device programming are essential to ensure accurate diagnosis and optimal management in pacemaker patients presenting with exertion-related symptoms.

## Lead author biography


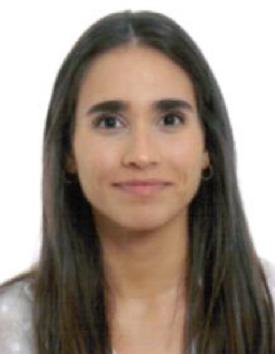


Clara Nuevo-Gallardo is currently a resident physician in Cardiology at University Hospital of Badajoz, with a particular interest in interventional cardiology.

## Data Availability

The data underlying this article was gathered from our institution’s database. It can be shared upon request to the authors, previous patient’s agreement.

## Notes

**Consent:** The patient has provided consent to publish this case report in accordance with COPE guidelines.
